# Composition and dynamics of the bacterial communities present in the post-slaughter environment of farmed Atlantic salmon (*Salmo salar* L*.*) and correlations to gelatin degrading activity

**DOI:** 10.7717/peerj.7040

**Published:** 2019-06-04

**Authors:** Ása Jacobsen, Svein-Ole Mikalsen, Hóraldur Joensen, Jonhard Eysturskarð

**Affiliations:** 1 Fiskaaling, Aquaculture Research Station of the Faroes, Við Áir, Hvalvík, The Faroe Islands; 2 Department of Science and Technology, University of the Faroe Islands, Tórshavn, The Faroe Islands

**Keywords:** Aquaculture, Atlantic salmon, Bacterial communities, Gelatinase activity, Post-slaughter

## Abstract

**Background:**

Microbial analyses performed in connection with the post-slaughter environment of farmed Atlantic salmon (*Salmo salar* L.) have mostly focused on specific bacteria that may have negative effects on the health of consumers. However, bacteria may also affect other quality variables. The objective of this study was to provide general knowledge about composition and dynamics of the bacterial communities present at slaughter and cold storage of farmed Atlantic salmon, as well as reveal any possible correlations to gelatinase activity, which may affect fillet quality. Thus, these data may provide a basis for optimization opportunities in the aquaculture industry.

**Methods:**

Samples were taken from the digestive system harvested from 15 salmon immediately after slaughter. Another 17 salmon were taken from the processing line just before the final cleaning stage; of these eight were distributed in three iced storage boxes while the other nine were rinsed an extra time with industrial water before being distributed into another three storage boxes. In the following 6 days, samples were taken of skin mucus, liquids in the abdominal cavity and the storage ice. The compositions of the bacterial communities were analyzed by next-generation sequencing and gelatinase activity was measured in all samples except the storage ice.

**Results:**

The bacterial communities in the digestive tract samples were dominated by the family *Mycoplasmataceae.* The genus *Aliivibrio* was also relatively abundant. Bacterial communities in the abdominal cavity were generally more diverse than the intestinal samples. However, all of the abdominal samples from storage box no. 3 had a high relative abundance of *Mycoplasmataceae*, and could not be distinguished from the intestinal samples (*Q* = 1.27, *p* = 0.633) while being significantly different from the other abdominal samples (*Q* = 9.02, *p* = 0.01). In addition, the abdominal samples from storage box no. 3 had a significantly higher gelatin degrading activity (*Q* = 9.43, *p* = 0.001) than those from the other storage boxes and similar to the high gelatinase activity in the intestinal samples. This indicated that in storage box no. 3 there was a transfer of intestinal fluids to the abdominal cavities, which was not removed by the cleaning procedure. There was a significant difference of the major phyla detected in the skin mucus of salmon rinsed an additional time, as these salmon had a higher relative amount of *Firmicutes* (*F* = 4.76, *p* = 0.04) and lower amount of *Proteobacteria* (*F* = 4.41, *p* = 0.047).

**Conclusions:**

The study showed a correlation between intestinal fluids and bacteria left in the abdominal cavity and gelatinase activity. This suggested that intestinal fluids and/or bacteria could enhance the degradation of connective tissue in the abdominal cavity and hence negatively affect the fillet quality. In addition, the study provided general knowledge of the composition and dynamics of bacterial communities present.

## Introduction

The post-mortem degradation of connective tissue in Atlantic salmon (*Salmo salar* L.) fillets leading to lower quality has mainly been attributed to the enzymatic activity of matrix metalloproteinases (MMPs) (Pedersen et al., 2015). MMPs are excreted by various cells in the soft and hard connective tissues (Verma & Hansch, 2007), and are therefore present in the tissue of slaughtered fish in cold storage even after having been bled out. However, a recent study (Jacobsen, Joensen & Eysturskarð, 2017) found that blood and other bodily fluids or remains left in the abdominal cavity during cold storage had a significant effect on the degree of gaping and soft fillets in Atlantic salmon. This suggests that MMPs and enzymes other than those inherently present in the muscle tissue can be damaging to the connective tissue during cold storage of the salmon. High concentrations of several MMPs have been measured in salmon blood (Eysturskarð et al., 2017) and MMPs have also been reported in bile of other fish species (Hauser-Davis, Lima & Campos, 2012). In addition to MMPs produced by the salmon itself, many bacteria also produce MMPs and other collagenolytic proteases (Zhang et al., 2015; Duarte, Correia & Esteves, 2016) as well as proteolytic enzymes that can activate host proMMPs (Okamoto et al., 1997). Bacterial collagenases or gelatinases (MMP subfamilies) not directly associated with pathogenic activity have also been isolated from various fish species and their surroundings after slaughter (Sai-Ut, Benjakul & Sumpavapol, 2013). Previous analysis of bacteria present in the slaughtering and processing environment of farmed salmon or other farmed fish species have mostly focused on specific spoilage bacteria and the methods have often been culture dependent (Morey, Himmelbloom & Olivieira, 2014; Langsrud et al., 2015). The general composition and dynamics of the bacterial community present on or in the salmon and its cold-storage environment are more seldomly reported, although some recent analyses have been made (Reynisson et al., 2010; Møretrøet al., 2016). Here we have made concurrent analyses of the gelatin degrading potential and the bacterial community in the digestive system at slaughter and in the skin mucus, and fluids from the abdominal cavity over a period of seven days. In addition, the bacterial community composition in the storage ice was also investigated. This has resulted in an improved understanding of the potential correlations between external fluids and connective tissue degradation in the fillets. Furthermore, the information gained about the bacterial compositions in the post-slaughter environment of salmon is a valuable addition to the basic knowledge of the bacterial communities on and in salmon.

## Materials & Methods

### Samples

**Figure 1:**
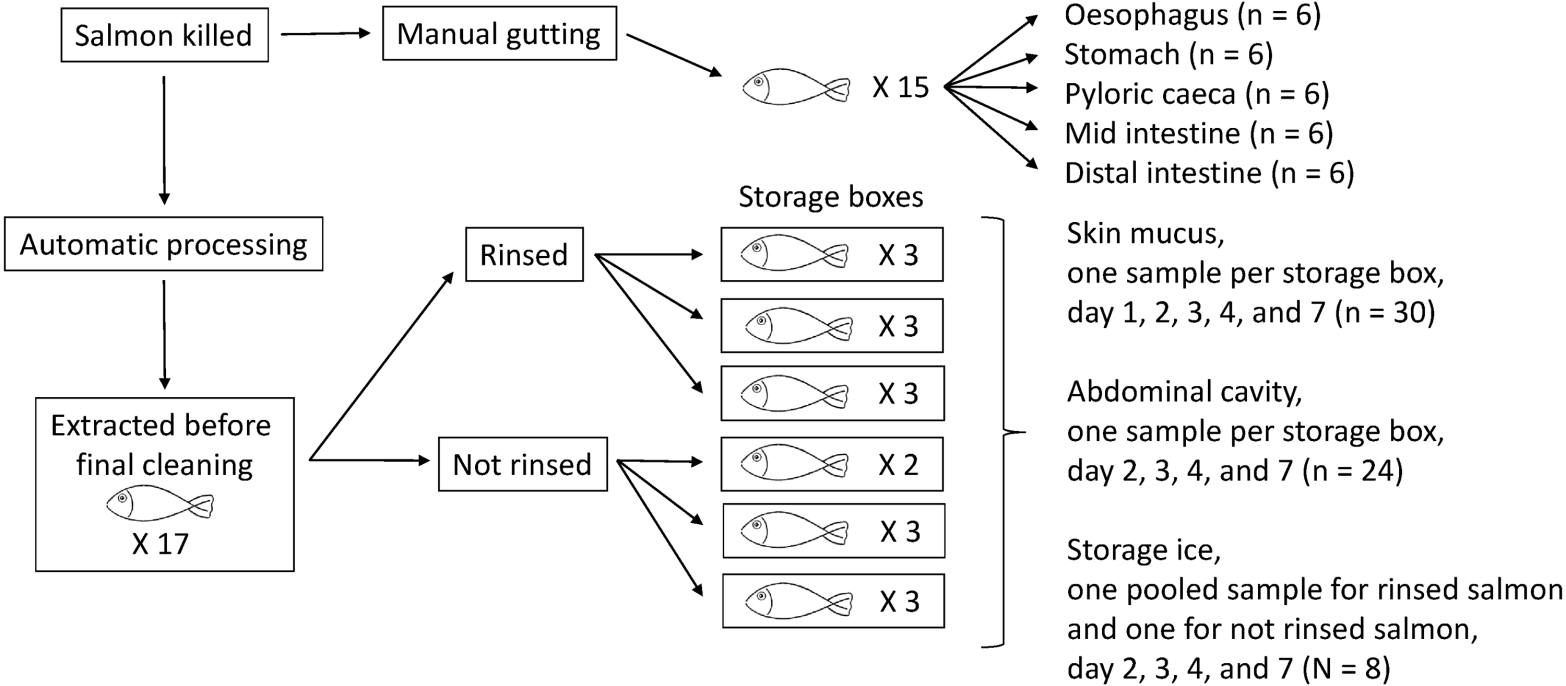
Illustration of experimental setup. Fifteen salmon were gutted manually immediately after being killed following standard procedure. Intestines were harvested and frozen for sampling at a later stage. The same day another 17 salmon were taken of the proceesing table just prior to the final cleaning stage. These salmon were distributed into six storage boxes with storage ice as standard procedure. Salmon in three of the storage boxes were rinsed manually with industrial water before being stored while salmon in the other three storage boxes were not. All storage boxes were placed in the cooling facility (∼2.0 °C) as standard storage procedure and all sampling was performed there without taking the salmon out of the storage boxes.

The entire digestive system was harvested from 15 salmon at slaughter in a processing facility owned by the local farming company P/F Bakkafrost (Glyvrar, The Faroe Islands). Whole intestines were taken from the abdominal cavity of the salmon when gutted and put in sterile plastic bags and immediately stored in dry ice in closed containers. The containers were transported to the laboratory within a few hours and the bags with digestive systems stored at −80 °C until the experimental setup was completed approximately a week later. Prior to sampling, the bags containing the digestive systems were taken out of the freezer and left in a refrigerator (4 °C) to slowly thaw overnight. At sampling the digestive systems were still chill and frozen but manageable. Samples were taken from the distal intestine (DI), mid intestine (MI), pyloric caeca (Py), stomach (St) and oesophagus (Oe). Sterile scalpels were used to open the organs while using another sterile scalpel to carefully scrape out both content and wall mucus without scraping off organ material. Because the salmon, according to standard procedure, were starved for a few days before slaughter, limited amount of material was expected in the digestive tract. Where possible, 1 mL of material per sample was used from each salmon and materials from two salmon were since pooled into one 2 mL sample. If one of the salmon did not contain enough material a third salmon was used as supplement. In total there were six pooled samples per digestive tract location. See Fig. 1 for illustration of the experimental setup and sampling procedure. DNA extraction was performed immediately following sampling. Overall, the sampling procedure of the digestive system samples were designed to eliminate possible DNA degradation or alteration of the microbial composition (Tedjo et al., 2015).

In order to simulate standard storage and transport conditions for the slaughtered salmon, another group of 17 salmon were distributed into six storage boxes and sampled several times during cold storage (Fig. 1). These salmon were removed from the standard processing line just before the final cleaning operation in order to investigate the bacterial communities present with two different cleaning conditions. Two or three salmon were stored in each standard storage box and covered with ice of industrial (filtered and UV treated) water. The salmon in replicate storage boxes no. 1, 2, and 3 were manually rinsed once more with the same filtered and UV treated water (also used in the previous cleaning operations in the processing line) before being stored, while the salmon in replicate storage boxes no. 4, 5, and 6 did not go through the extra rinse. The storage boxes were thereafter placed in the cooling facility with a temperature of approximately 2.0 °C according to standard storage procedure. All sampling was performed in the cooling facility without taking the salmon out of the boxes.

Samples of skin mucus (samples abbreviated “S”) were taken on day 1, 2, 3, 4 and 7. Samples of mucus and liquid from the abdominal cavity (samples abbreviated “B”) were taken on same occasions, except on day 1. Sterile scalpels were used to scrape of mucus and other liquids and remains from the two sampling sites. Care was taken not to puncture or otherwise damage the skin or inner lining of the salmon during sampling . The skin mucus sampled from all salmon in a storage box were pooled resulting in one sample per storage box per sampling day. Likewise for the abdominal samples. At the same time as abdominal samples were taken, 100 mL of slush ice (samples “K”) was sampled from the bottom of each storage box. The ice from storage boxes no. 1, 2, and 3 were pooled into one sample for each sampling day and the same with the ice from storage boxes no. 4, 5, and 6 (Fig. 1).

Because conditions at the cooling facility were not appropriate for performing homogenization and aliquoting, all samples were put on dry ice immediately at sampling and since stored at −80 °C until further processing and analyses were performed. Deep freeze storage for a short period has been shown to have only minor effect on enzyme activity measurements and bacterial community analysis (Wallenius et al., 2010). Even two freeze thaw cycles prior to enzymatic measurements was estimated to have minor influence based on published experiments (Murias, Rachtan & Jodynis-Liebert, 2005; Cuhadar et al., 2013). When the experimental setup was finished, samples were thawed and homogenized by pipetting such that analyses of both bacterial community and enymatic activity could be performed of the same sample. Subsamples from the homogenized samples were taken for performing the sequencing protocol while the remainder of the sample was used for measurements of gelatinase activity. If analysis of the gelatinase activity could not be done in parallel to the bacterial community analyses the samples were re-frozen until those analyses could be performed later, within a few days.

### DNA extraction

There were 96 samples in total (Fig. 1) that were subjected to DNA extraction. Samples from the digestive system were processed immediately after sampling. Samples were mixed until homogenous, and from these, subsamples of 220 mg were taken for DNA extraction using the QIAamp Stool mini kit (Qiagen, Hilden, Germany) while the remainings of the samples were frozen and stored at −80 °C until further analysis. The DNA extraction was performed according to the extraction kit protocol.

The ice from the storage boxes was thawed at room temperature and filtered using 0.22 µm filters. The 100 mL of ice from each of the storage boxes no. 1–3 from the same sampling days were filtered together and thus pooled into one sample while the storage ice from boxes no. 4–6 were pooled into one sample for each sampling day. DNA was then extracted from the filters using the PowerWater DNA extraction kit following the supplier’s instructions (Qiagen).

Skin mucus and abdominal samples from all sampling days except day 3 were extracted using the PowerSoil DNA extraction kit (Qiagen) following the manufacturer’s protocol. The PowerSoil DNA extraction kit is extensively used in metagenomics although it does not give high yield in comparison with other methods (Vishnivetskaya et al., 2013; Rubin et al., 2014). However, in a comparison of extraction methods and the subsequent results of sequencing on the Illumina MiSeq platform, the relatively low DNA concentration achieved in the initial extraction did not seem to have any substantial negative effect on the number of OTUs achieved and diversity measurements of the bacterial community (Burbach et al., 2016). For comparison, samples from day 3 were extracted using the DNeasy Blood and Tissue kit (Qiagen), which also has been used extensively for 16S rRNA sequence analysis, by following the protocol for pretreatment of gram negative and gram positive bacteria for two subsamples and combining the subsamples in step 4 in the supplier’s protocol “Purification of total DNA from Animal Tissues”. The DNA concentration was measured in all samples using the Quant-iT PicoGreen dsDNA Assay kit (Thermo Fisher Scientific, Waltham, MA, USA) and a Glomax Multi+ Detection System (Promega Biotech AB, Nacka, Sweden).

### Sequencing

Library preparation was performed according to the Illumina “16S Metagenomic Sequencing Library Preparation” document (Part # 15044223 Rev. B) with minor modifications using the recommended universal amplicon primers (selected from Klindworth et al., 2013) covering the V3 and V4 regions for the first round PCR. The primer sequences including the Illumina overhang adapters were: 5′-TCGTCGGCAGCGTCAGATGTGTATAAGAGACAGCCTACGGGNGGCWGCAG-3′ and 5′-GTCTCGTGGGCTCGGAGATGTGTATAAGAGACAGGACTACHVGGGTATCT AATCC-3′ for the forward and reverse primers, respectively. The PCR mix contained 5 µl of 1 µM forward and reverse primers each, 12.5 µl of KAPA HiFi HotStart Ready Mix (Roche Diagnostics, Rotkreuz, Switzerland), and 2.5 µl sample DNA. The DNA concentration used for the amplicon PCR was approximately 6 ng/µl instead of the recommended 5 ng/µl. The concentration was increased due to the probability of host DNA presence. The thermocycling conditions were: 95 °C for 3 min, then 26 cycles of 95 °C for 30 s, 55 °C for 30 s, and 72 °C for 30 s, followed by 72 °C for 5 min and final hold at 4 °C. Number of cycles in the amplicon PCR was increased from the recommended 25 to 26 because the DNA concentration was estimated to otherwise be too low. PCR products from representative samples for each sample types were run on a BioAnalyzer using the High Sensitivity DNA kit (Agilent, Santa Clara, CA, USA) to verify the size and purity before continuing analysis. After verification, the index PCR was performed using primers from the Illumina Nextera XT Index kit. The thermocycling conditions were the same as with the initial amplicon PCR, but with only 10 cycles this time. Representative samples were then run on the BioAnalyzer to verify the size and purity of the libraries. The libraries were measured for DNA concentration using the Quant-iT PicoGreen dsDNA Assay kit (Thermo Fisher Scientific). The final pooled library loaded into the MiSeq instrument for sequencing had a concentration of 5 pM containing 6.67% PhiX control.

### Data analysis

Fastq files were downloaded from the BaseSpace Sequence Hub and analysed in QIIME (Caporaso et al., 2010a). Quality score plots of assembled and unassembled R1 and R2 reads after joining the paired end reads using the SeqPrep (https://github.com/jstjohn/SeqPrep) and fastq-join (Aronesty, 2013) methods were compared. SeqPrep performed better than fastq-join in this instance and was used for assembling all reads. The assembled reads with a minimum average quality score of Q30 were further quality filtered and sorted into samples by the *split_libraries-fastq.py* command (Caporaso et al., 2012) using the default values for quality thresholds. This resulted in the removal of single end reads with less than 75% consecutive high quality base calls and unassigned reads, as well as the truncation of reads with more than three consecutive low quality base calls. ChimeraSlayer was applied before OTU picking and did not detect any chimeras. The workflow command *pick_de_novo_otus.py* was applied to cluster the reads into OTUs with 97% similarity by the *de novo* method, representative reads were aligned with PyNAST (Caporaso et al., 2010b), and taxonomy was assigned using the UCLUST method (Edgar, 2010). A phylogenetic tree was also constructed with the program FastTree (Price, Dehal & Arkin, 2009) and finally an OTU table was produced. All OTUs with less than five reads were removed. Removal of low abundance OTUs has also been shown to reduce the content of chimeras substantially (Majaneva et al., 2015; Auer et al., 2017) compensating for possible failure of ChimeraSlayer to detect chimeras (Majaneva et al., 2015). The within sample diversity was analyzed using the *alpha_rarefaction.py* command, calculating the alpha diversity metrics Chao1 (Chao, 1984), observed OTUs, and PD whole tree (Faith, 1992). Calculations of the between samples diversity was made using the *beta_diversity_through_plots.py* including the phylogenetic tree and 4,000 reads per sample. The command produced a weighted UniFrac (Lozupone & Knight, 2005) distance matrix and a principle coordinates file that was visualised using the *make_emperor.py* command (Vázquez-Baeza et al., 2013). Bacterial communities were reported at phylum level and at the most specific taxonomic rank achieved from the analysis.

### Multivariate analysis

In order to get a comprehensive evaluation of the sequencing data, a data matrix was subjected to principal component analysis (PCA) (Wold, 1979) using the software package SIRIUS (Kvalheim & Karstang, 1987). The objects were all successfully sequenced samples (*n* = 73) and the variables were all the different bacteria taxa detected and presented in the OTU table (*n* = 365). Before PCA, the variables were centered by subtracting their means and the objects were block normalized and log-transformed. These transformations warrant proper comparison of the objects and ensure appropriate influence of the variables, large or small. During PCA, the objects were placed in a multi-dimensional vector space, one coordinate for each variable. New orthogonal coordinates, the principal components (PCs) were then generated through the centroid of all samples in the multidimensional space in the direction of the largest and second largest and the third largest dispersion of the objects. In this way, the relationship among the objects could be depicted in only two and three dimensions without substantial loss of the total variance.

### Measurements of gelatinase activity

The EnzChek® Gelatinase/Collagenase Assay Kit (Thermo Fisher Scientific, Waltham, MA, USA) was used to screen the various samples for potential gelatinase/collagenase activity. DQ gelatin (20 µl of 100 µg/mL) was used as substrate and 50µL of homogenized sample was mixed with 50 µL of 1x buffer and added to each well. A negative control containing only substrate and 1x buffer and a positive control containing the *Clostridium* collagenase supplied with the kit were run with every plate measured. All samples were run in duplicates. The samples were measured in a Glomax Multi+ Detection System at ex/em = 490/510−570 nm every 10 min over a period of 15 h. The background fluorescence measured in the negative controls was subtracted from all samples to achieve the Relative Fluorescence Unit (RFU).

### Statistics

Significant differences in the alpha diversity estimates and relative content of specific OTUs between two sample groups were tested with ANOVA (F-statistic). Comparisons between three or more groups was in addition analysed by the Tukey HSD (Q-statistic) for groups with unequal number of replicates (Kramer, 1956) as implemented in the online calculator (http://astatsa.com/OneWay_Anova_with_TukeyHSD/). Significance of beta diversity between sample types was tested with PERMANOVA performed in QIIME. Significance was accepted at *p*-values < 0.05.

### Ethics statement

This study complied with the boundaries of EU legal frameworks relating to the protection of animals used for scientific purposes (i.e., Directive 2010/63/EU). No specific permit was needed since the industrial procedures in capture and slaughter were followed, and none of these parts were initiated or altered due to this study. Tissue sampling took place post-mortem following standard procedures performed by the local aquaculture industry, authorized by the Faroese Ministry of Foreign Affairs and Trade.

## Results

### 16S rRNA sequencing

The sequencing process resulted in 22,231,798 paired end reads in total. After paired end sequences were joined and quality filtered, a total of 4,869,297 high quality reads were available for analysis. After removal of the OTUs supported by less than five reads in total, the number of reads was 4,814,980. Seventy three samples were successfully sequenced. Most of the samples with insufficient number of reads for analysis (<1,000 reads) were from the upper gastrointestinal area. In addition five of the skin mucus samples taken on day 1 and 2 were not successfully sequenced. The 73 samples successfully sequenced are listed in Table 1 with description of sample type, sampling day, number of reads and OTUs.

**Table 1:** Sample information and number of reads and OTUs obtained. All organic samples were pooled from 2–3 individuals. Ice from storage boxes no. 1, 2, and 3 was pooled and ice from storage boxes no. 4, 5, and 6 was pooled. Alpha diversity estimates (displayed in Fig. 2) were based on 10 iterations using 16,018 rarefied reads for most samples, 10,682 reads ^†^ or 5,346 reads ^††^ for a few samples and one sample was excluded ^†††^.

**Sample type—day**	**Sample name**	**No. of reads**	**No. of OTUs**	**Sample type—day**	**Sample name**	**No. of reads**	**No. of OTUs**
Stomach—1	ST-5	96,776	851	Abd. Fluids—7	B4-day 7	40,291	960
Pyloric caeca—1	PY-1	53,549	629	Abd. Fluids—7	B5-day7	53,371	1,210
Pyloric caeca—1	PY-2	31,223	417	Abd. Fluids—7	B6-day7	46,045	712
Pyloric caeca—1	PY-3	87,905	812	Skin mucus—1	S3-day1	36,139	469
Pyloric caeca—1	PY-4	113,453	923	Skin mucus—1	S4-day1	93,522	1,278
Pyloric caeca—1	PY-5	81,104	750	Skin mucus—1	S5-day1	62,329	973
Pyloric caeca—1	PY-6	95,580	843	Skin mucus—1	S6-day1	77,240	932
Mid intestine—1	MI-2	122,253	723	Skin mucus—2	S3-day2	5,155	526^†††^
Mid intestine—1	MI-3	42,600	546	Skin mucus—2	S4-day2	5,925	514^††^
Mid intestine—1	MI-4	117,836	904	Skin mucus—2	S5-day2	63,979	1,148
Mid intestine—1	MI-5	101,842	840	Skin mucus—2	S6-day2	119,630	1,343
Mid intestine—1	MI-6	129,901	928	Skin mucus—3	S1-day3	49,433	664
Distal intestine—1	DI-1	240,851	1,407	Skin mucus—3	S3-day3	55,544	706
Distal intestine—1	DI-3	84,371	399	Skin mucus—3	S4-day3	63,941	880
Distal intestine—1	DI-4	132,766	826	Skin mucus—3	S5-day3	50,891	805
Distal intestine—1	DI-6	86,550	789	Skin mucus—3	S6-day3	81,530	870
Abd. Fluids—2	B1-day2	6,300	508^††^	Skin mucus—4	S1-day4	17,318	972
Abd. Fluids—2	B2-day2	26,565	772	Skin mucus—4	S2-day4	5,859	417^††^
Abd. Fluids—2	B3-day2	237,438	893	Skin mucus—4	S3-day4	16,106	933
Abd. Fluids—2	B4-day2	41,085	775	Skin mucus—4	S4-day4	11,235	527^†^
Abd. Fluids—2	B5-day2	21,318	790	Skin mucus—4	S5-day4	26,064	753
Abd. Fluids—2	B6-day2	22,222	326	Skin mucus—4	S6-day4	14,008	452^†^
Abd. Fluids—3	B1-day3	28,587	806	Skin mucus—7	S1-day7	42,759	733
Abd. Fluids—3	B2-day3	45,885	908	Skin mucus—7	S2-day7	34,291	857
Abd. Fluids—3	B3-day3	85,806	672	Skin mucus—7	S3-day7	24,385	906
Abd. Fluids—3	B4-day3	21,631	767	Skin mucus—7	S4-day7	20,006	388
Abd. Fluids—3	B5-day3	20,031	1,080	Skin mucus—7	S5-day7	62,777	798
Abd. Fluids—3	B6-day3	41,540	820	Skin mucus—7	S6-day7	21,771	734
Abd. Fluids—4	B1-day4	62,987	985	Storage ice—2	K123-day2	54,001	690
Abd. Fluids - 4	B2-day4	14,790	600^†^	Storage ice—2	K456-day2	34,806	558
Abd. Fluids—4	B3-day4	343,692	1,145	Storage ice—3	K123-day3	95,613	795
Abd. Fluids—4	B4-day4	34,765	764	Storage ice—3	K456-day3	69,222	803
Abd. Fluids—4	B5-day4	22,145	974	Storage ice—4	K123-day4	79,536	756
Abd. Fluids—4	B6-day4	17,807	875	Storage ice—4	K456-day4	20,499	451
Abd. Fluids—7	B1-day7	66,712	1,193	Storage ice—7	K123-day7	91,708	784
Abd. Fluids—7	B2-day7	90,507	1,139	Storage ice—7	K456-day7	90,332	598
Abd. Fluids—7	B3-day7	207,346	1,016				

The minimum and maximum number of reads per samples was 5,155 and 343,692, respectively. The average and median number of reads per sample was 65,959 and 53,371, respectively. The average number of OTUs per sample was 798.5. The individual rarefaction curves (Fig. S1) indicated sufficient, but not saturating, sequencing depths for most samples while a few of the samples with number of reads below approximately 25,000 would benefit from a higher number of reads. Group-wise rarefaction curves based on sample types are illustrated in Fig. S2.

### Alpha diversity

**Figure 2:**
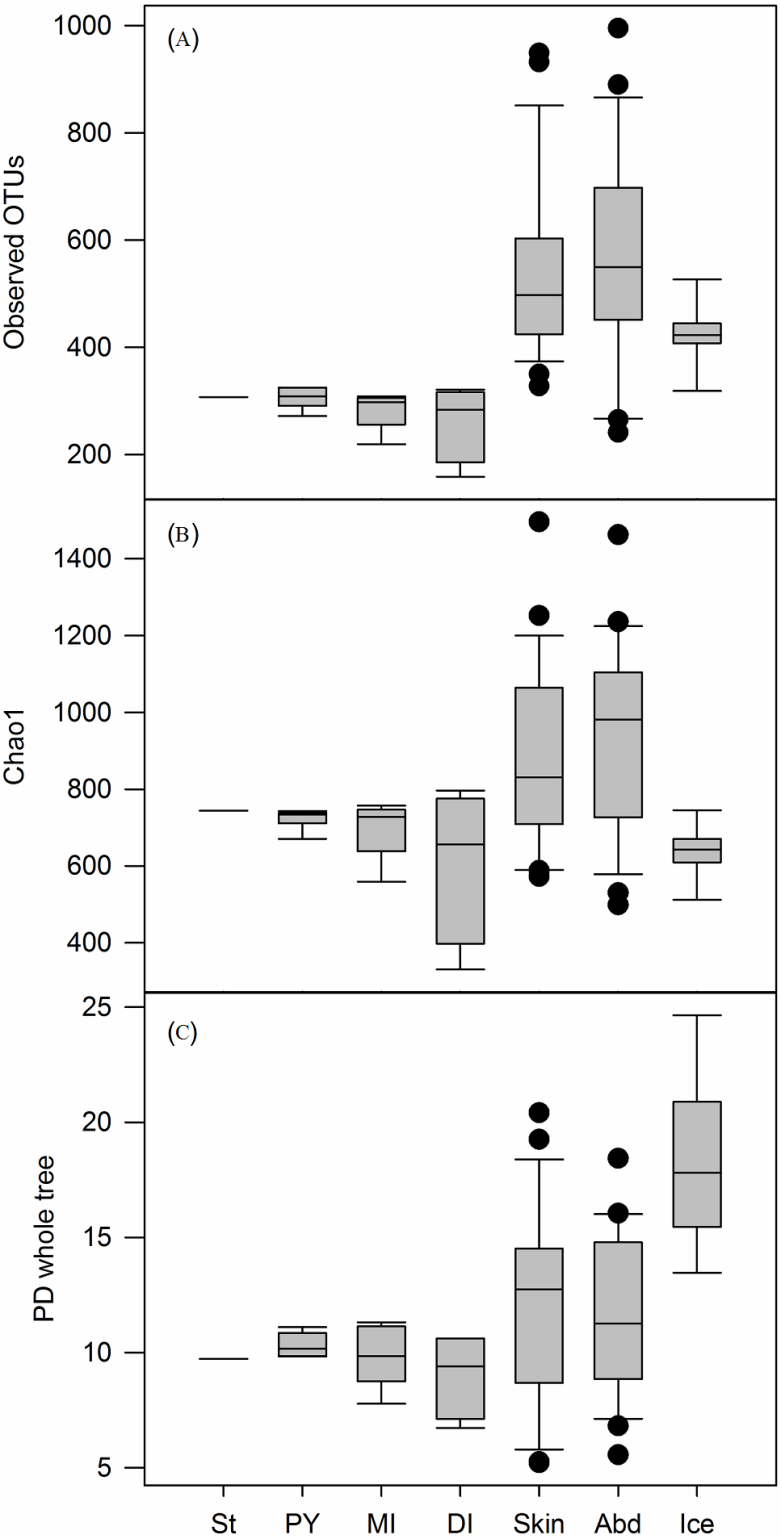
Alpha diversity estimates for all samples types. (A) Observed OTUs, (B) Chao1, and (C) PD whole tree estimates for stomach (St, *n* = 1), pyloric caeca (PY, *n* = 6), mid intestine (MI, *n* = 5), distal intestine (DI, *n* = 4), skin mucus (S, *n* = 25), abdominal fluids (B, *n* = 24) and storage ice (K, *n* = 8). Boxes indicate median and 1st and 3rd quartile. Whiskers indicate standard deviations and dots represent outliers. Samples and iterations for alpha diversity estimates are described in Table 1.

OTU richness was estimated by several alpha diversity metrics: (i) observed OTUs, which is the number of OTUs detected by subsampling every sample several times at a standardised sequencing depth; (ii) Chao1, which adds a correction factor taking into account the low abundance OTUs, and (iii) the phylogenetic diversity estimate PD whole tree, which calculates the branch lengths in the phylogenetic tree constructed from each sample. Within the salmon digestive tract samples, the pyloric caeca had the highest OTU richness estimates (Figs. 2A and 2B) and phylogenetic diversity (Fig. 2C), followed by the mid intestine and then the distal intestine, but these differences were not statistically significant (Obs. OTUs: *F* = 1.22, *p* = 0.33; Chao1: *F* = 1.3, *p* = 0.31, PD wt: *F* = 1.2, *p* = 0.335). The alpha diversity estimates for the single stomach sample were within the range of the estimates for the other digestive tract samples. However, because only one sample was available for the stomach, it was not included in this or other statistical analyses.

OTU richness and phylogenetic diversity in the skin mucus and abdominal fluids were at a similar level and there was a great deal of variation between individual samples for both sample types (Fig. 2). The skin mucus and abdominal fluids had a significantly higher observed OTU estimate than the digestive tract samples from pyloric caeca (*Q* = 45.46, *p* = 0.024; *Q* = 49.87, *p* = 0.01), mid intestine (*Q* = 46.19, *p* = 0.021; *Q* = 50.30, *p* = 0.009), and distal intestine (*Q* = 45.78, *p* = 0.022; *Q* = 49.52, *p* = 0.01) respectively. For Chao1, the distal intestine had significantly lower values than the abdominal samples (*Q* = 42.13, *p* = 0.045) while the storage ice samples had significantly lower values than both skin mucus (*Q* = 42.29, *p* = 0.044) and abdominal samples (*Q* = 50.67, *p* = 0.008). On the other hand, the storage ice had significantly higher phylogenetic diversity values than all the other sample types (PY: *Q* = 59.08, *p* = 0.001; MI: *Q* = 58.75, *p* = 0.001; DI: *Q* = 60.58, *p* = 0.001; S: *Q* = 59.74, *p* = 0.001; B: *Q* = 65.96, *p* = 0.001).

### Bacterial community compositions

#### The digestive tract

**Figure 3:**
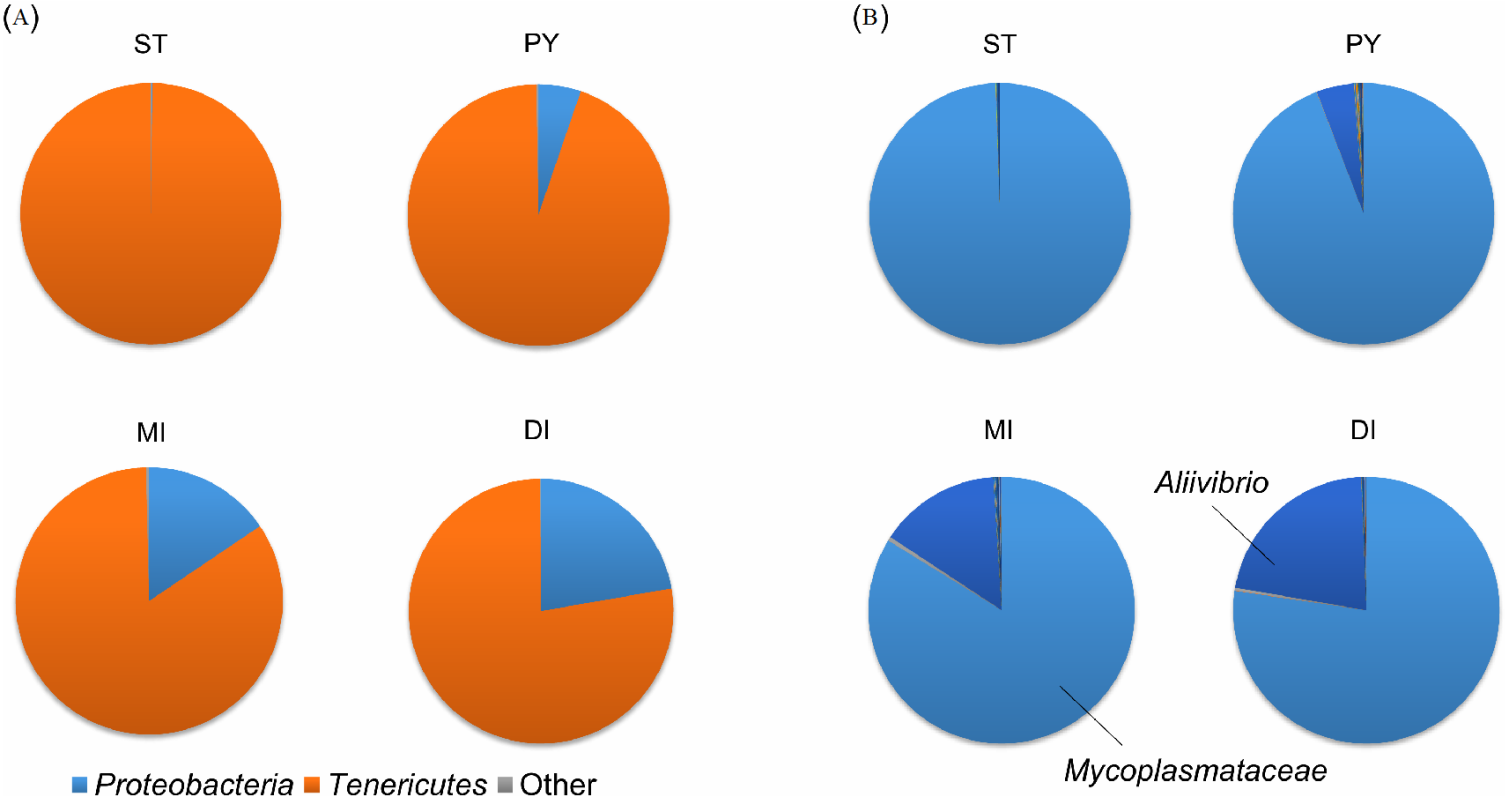
Composition of the bacterial community in the digestive system. Composition of the bacterial community at (A) phylum and (B) genus level. Each sample was pooled from 2–3 salmon. The graphs represent the averages of the various sample types. ST, Stomach (*n* = 1), PY, Pyloric caeca (*n* = 6), MI, Mid intestine ( *n* = 5), DI, Distal intestine (*n* = 4). Main phyla and genera are indicated.

The phylum *Tenericutes* was very dominating in the digestive tract, representing between 77.6% and 99.8% of the OTUs detected (Fig. 3A).

The phylum *Proteobacteria*, which is often detected in the intestines of salmon (Gajardo et al., 2016; Dehler, Secombes & Martin, 2017), was nearly absent in the single stomach sample with only 0.2% but was in average increasingly more abundant further down the digestive system and represented 22.3% of the OTUs detected in the distal intestines. However, the ANOVA/Tukey HSD Statistical test comparing the three sample groups (excluding the stomach sample) detected no significant difference (*F* = 1.0078, *p* = 0.39). The salmon digestive tract samples contained a bacterial community structure highly dominated by one single or two OTUs. *Mycoplasmataceae* of the phylum *Tenericutes* was the overall most dominant bacterial family represented in the digestive tract samples with between 77.3% and 99.4% (Fig. 3B). The genus *Aliivibrio* belonging to the family *Vibrionaceae* of the phylum *Proteobacteria* was also well represented in the samples, especially from the distal intestines where it represented 21.6% of the OTUs.

### The abdominal fluids

**Figure 4:**
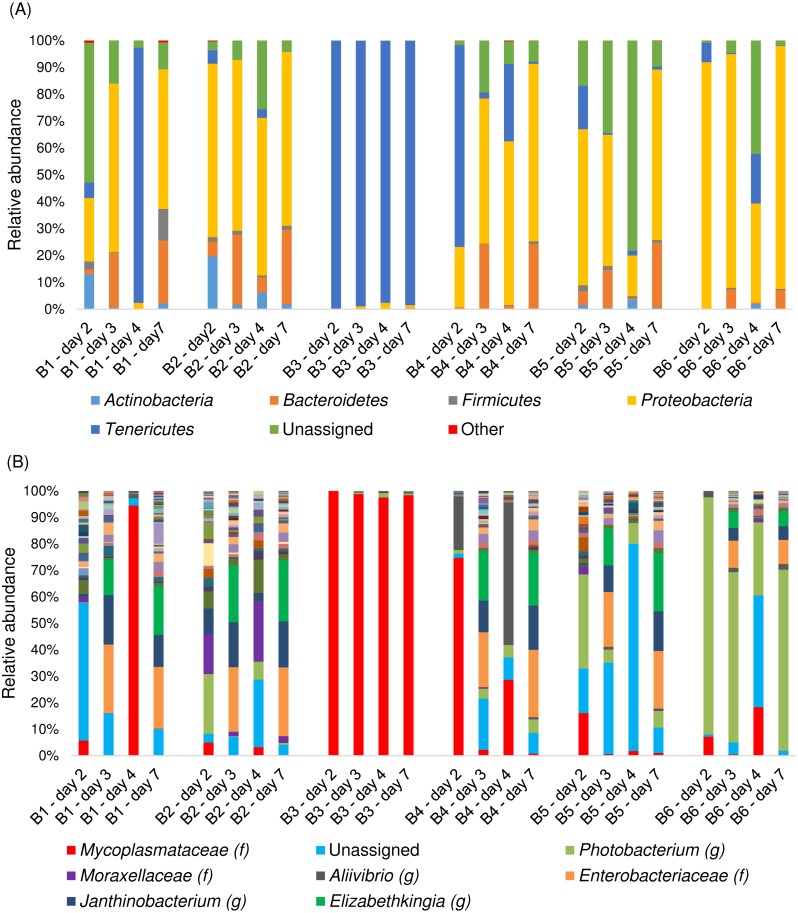
Composition of the bacterial community in samples from the abdominal cavity. Composition of the bacterial community at (A) phylum and (B) genus level. The six groups of columns represent the four sampling days from storage boxes no. 1–6. Each column is a pooled sample from the 2–3 salmon in each storage box. f, family, g, genus.

The compositions of the bacterial communities in the abdominal samples taken from salmon in all storage boxes except no. 3 were relatively similar (Fig. 4).

In the abdominal samples from these five storage boxes, *Proteobacteria* was the dominating phylum. There was also a high representation of *Bacteroidetes*, and on some occasions *Tenericutes* (Fig. 4A). In contrast, the abdominal samples taken from salmon in storage box no. 3 were highly dominated by the phylum *Tenericutes* with between 97.5% and 99.9% of the reads. The Tukey HSD test showed that the abdominal samples from storage box no. 3 had a significantly higher relative content of *Tenericutes* than the abdominal samples from the other storage boxes (B1: *Q* = 6.07, *p* = 0.005; B2: *Q* = 7.98, *p* = 0.001; B4: *Q* = 5.94, *p* = 0.006; B5: 7.75, *p* = 0.001; B6: *Q* = 7.61, *p* = 0.001). All abdominal samples from storage box no. 3 consisted almost entirely of the family *Mycoplasmataceae* (Fig. 4B), in contrast to the other abdominal samples (*Q* = 9.02, *p* = 0.01) which generally had a higher bacterial diversity and had similar relative contents of *Mycoplasmataceae* (*F* = 0.80, *p* = 0.544). On the other hand, there was no significant difference detected between the relative content of *Mycoplasmataceae* in the abdominal samples from storage box no. 3 and the digestive tract samples (*Q* = 1.27, *p* = 0.633). In storage box no. 6 there was a relatively high representation of the genus *Photobacterium* (fam. *Vibrionaceae*) ranging between 27.6% and 89.6% of the reads obtained the various sampling days (Fig. 4B). This genus was detected at significantly lower abundances in some of the other samples from the other storage boxes (*F* = 13.93, *p* < 0.001). Members of the family *Enterobacteriaceae* were detected in all storage boxes, particularly on sampling days 3 and 7, with up to 25.9%. *Janthinobacterium* (fam. *Oxalobacteriaceae*) and *Elizabethkingia* (fam. *Flavobacteriaceae*) were also detected at highest relative abundance on these two sampling days. No significant differences were detected between the storage boxes for these three bacteria (*F* = 0.78, *p* = 0.578; *F* = 1.57, *p* = 0.219; *F* = 0.86, *p* = 0.525).

### The skin mucus

**Figure 5:**
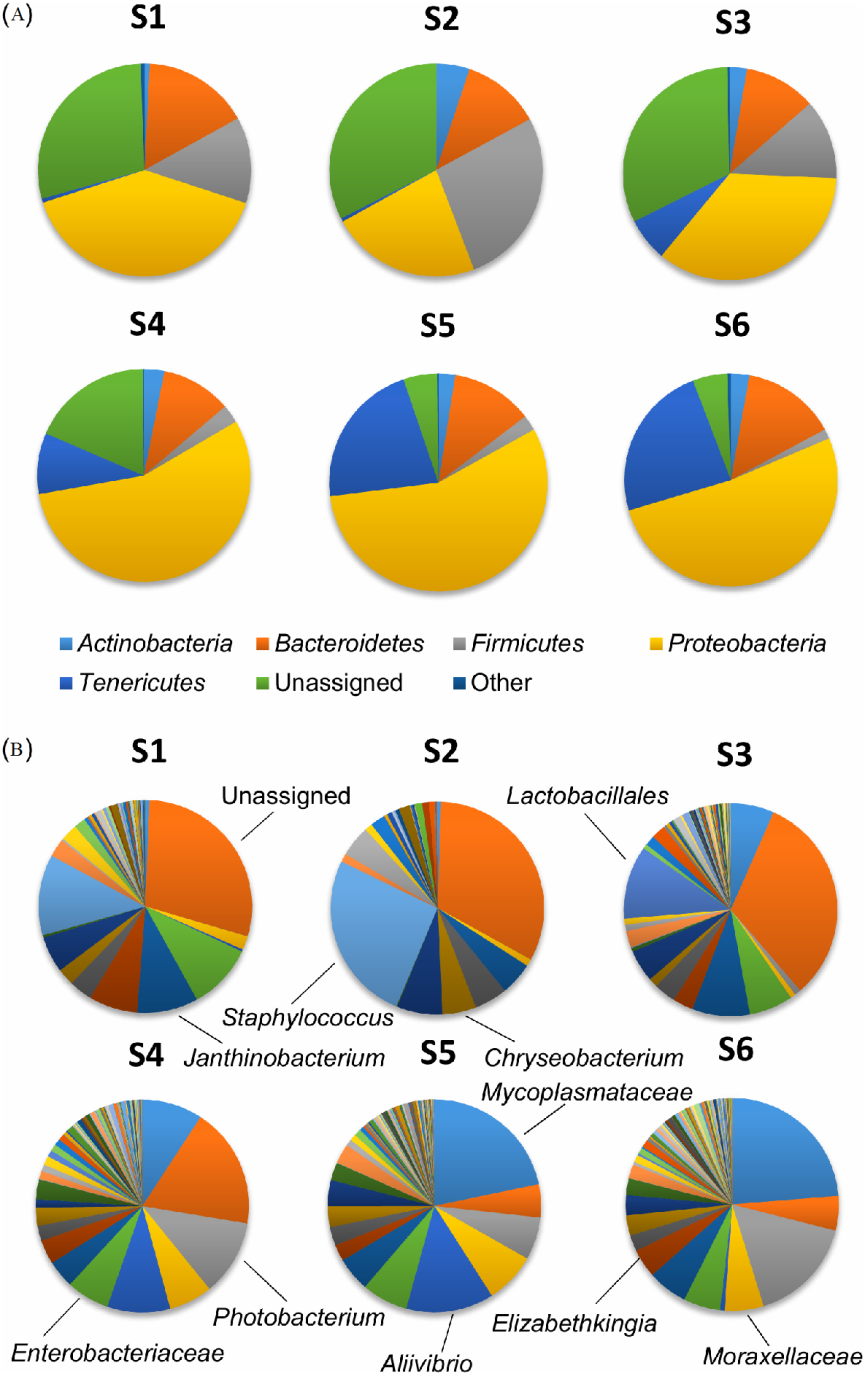
Composition of the bacterial community in the skin mucus. Composition of the bacterial community at (A) phylum and (B) genus level. Each pie represents the average of all sampling days from a storage box. S1 (*n* = 3), S2 (*n* = 2), S3 (*n* = 5), S4 (*n* = 5), S5 (*n* = 5), S6 (*n* = 5).

The skin mucus microbiota in salmon from storage boxes no. 1, 2, and 3, which were rinsed an additional time compared to the other salmon, was dominated by *Bacteroidetes* with 10.9 - 16.2%, *Firmicutes* with 12.1–27.1% and *Proteobacteria* with 22.7–40.0% as well as containing a relatively large proportion of unassigned bacteria (Fig. 5A).

In comparison the skin mucus microbiota from the other three storage boxes contained a significantly higher proportion of *Proteobacteria*, 51.8–56.1% (*F* = 4.41, *p* = 0.047) and a significantly lower proportion of *Firmicutes* (*F* = 4.76, *p* = 0.04). On average, storage boxes no. 4, 5, and 6 also had a higher occurrence of *Tenericutes* with up to 23.8% compared to maximum 6.6% in the samples from storage boxes no. 1, 2, and 3, although the difference was not statistically significant (*F* = 3.61, *p* = 0.07). There were several relatively abundant OTUs in the skin mucus samples from all storage boxes. *Janthinobacterium, Chryseobacterium* and *Elizabethkingia* (both belonging to fam. *Flavobacteriaceae*) and *Enterobacteriaceae* were all relatively abundant in most storage boxes (Fig. 5B). *Staphylococcus* (fam. *Staphylococcaceae*) and Lactobacillales were only relatively abundant in skin mucus samples from a few of the storage boxes. *Mycoplasmataceae, Moraxellaceae,* and *Aliivibrio* were mainly detected in the storage boxes containing salmon not rinsed an extra time and the relative content of *Photobacterium* was significantly higher in these samples than in those from salmon rinsed an extra time (*F* = 5.46, *p* = 0.03). The salmon rinsed an extra time on the other hand had a larger proportion of unknown bacteria.

### The storage ice

**Figure 6:**
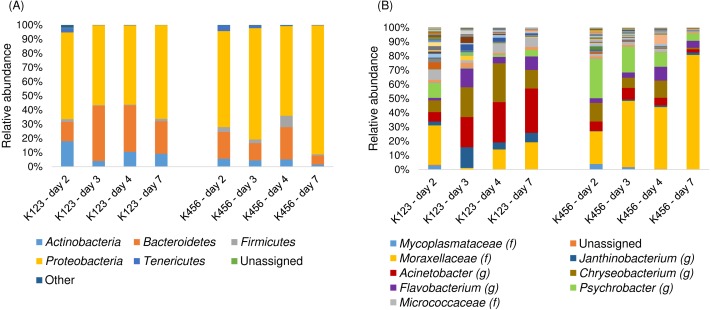
Composition of the bacterial community in samples from the storage ice. Composition of the bacterial community at (A) phylum and (B) genus level. The four bars on the left of both figures are the pooled samples from storage boxes 1–3 and the bars on the right represent the pooled samples from storage boxes 4–6. (A) The dominating phyla are illustrated in separate legends while those of very low abundance are combined in “Other” (B) The most abundant families (f) or genera (g) are illustrated in the legends.

*Proteobacteria* and *Bacteroidetes* were the most abundant phyla detected in the storage ice represented with between 55.9–90.9% and 6.2–39.0%, respectively (Fig. 6A), while *Actinobacteria* and *Firmicutes* were detected at lower levels. *Tenericutes* was detected at low levels the first sampling day, and further diminished in abundance over time.

On the first sampling day, there was a relatively even OTU abundance distribution, but over time the tendency was that a few OTUs became dominating while the low level OTUs represented a consistently decreasing part of the community (Fig. 6B). Ice from storage boxes no. 1–3, containing salmon that were rinsed an extra time, and ice from storage boxes no. 4–6 had a relatively similar bacterial community composition the first sampling day. However, over time the bacterial compositions seemed to change in two different ways. In storage boxes no. 4–6, the relative content of an unidentified bacterium in the family *Moraxellaceae* increased over the sampling period from 22.9% until it was a very dominant part of the bacterial community at 80.4%, while there was no increase on the relative content of *Moraxellaceae* in the storage ice from boxes no. 1–3. An ANOVA statistical comparison revealed a significant difference (*F* = 6.25, *p* = 0.047) in the content of *Moraxellaceae* between the storage ice from boxes no. 1–3 and boxes no. 4–6. *Acinetobacter*, another genus from the *Moraxellaceae* family increased from 6.7% to 31.2% in the ice from storage boxes no. 1–3 during the sampling period, which was significantly different (*F* = 8.39, *p* = 0.028) than the more constant relatively low abundance detected in the ice from storage boxes no. 4–6.

### Beta diversity

**Figure 7:**
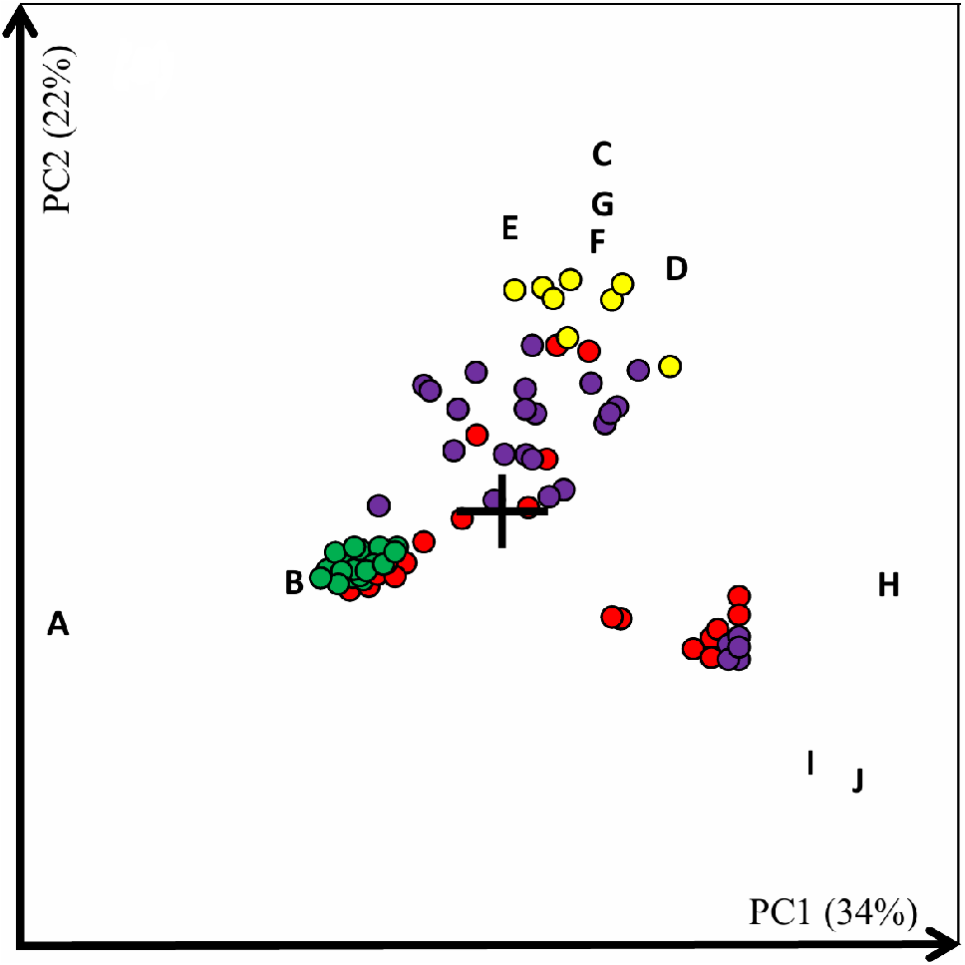
PC-plot of all samples based on variables comprising bacterial taxa. 2D PC-plot. Each circle represents one sample. Green, combined visceral samples including stomach, pyloric caeca, midgut and hindgut. Red, abdominal cavities. Violet, skin mucus. Yellow, storage ice. The first and second principal components (PC1 and PC2) describe 34% and 22%, respectively, of the total variance. The ten most discriminatory variables are illustrated. A, *Mycoplasmataceae* (f), B, *Aliivibrio* (g), C, *Moraxellaceae* (f), D, *Acinetobacter* (g), E, *Psychrobacter* (g), F, *Flavobacterium* (g), G, *Chryseobacterium* (g), H, *Janthinobacterium* (g), I, *Elizabethkingia* (g) and J, *Enterobacteriaceae* (f). f, family, g, genus.

All of the 73 samples and 10 of the most discriminating bacteria were displayed as objects and variables, respectively, in a PC1 versus PC2 coordinate system, resulting in a 2D plot (Fig. 7). Closely situated variables are positively correlated, while variables on either side of the origo are negatively correlated.

Variables positioned far away from the origo, marked as a cross in the 2D plot, had the largest influence on the placements of the samples in the plot. The variables with highest discriminating power were *Mycoplasmataceae* (A) and *Janthinobacterium* (H) for PC1 and *Moraxellaceae* (C) and *Enterobacteriaceae* (J) for PC2 (Fig. 7). The ten variables could effectively be sorted into four groups. Variables *Mycoplasmataceae* (A) and *Aliivibrio* (B), composing one such group, had a positive correlation with the grouped intestinal samples, marked in green, as would be expected by the dominating presence of these bacteria in those samples. Variables *Mycoplasmataceae* (A) and *Aliivibrio* (B) were also positively associated with seven samples from the abdominal cavity, which were inter-twined with the 15 samples from the digestive tract (Fig. 7). These abdominal samples included all four samples from storage box no. 3 as well as three others from various storage boxes. The second group with the positively correlated variables *Elizabethkingia* (I) and *Enterobacteriaceae* (J) were associated with some of the abdominal cavity and skin mucus samples which were drawn toward them in the right lower corner (Fig. 7). The variable *Janthinobacterium* (H), sole member of the third group, also influenced the positioning of these samples further to the right along the PC1 axis. However, the association between these two sample types was not as strong as between the intestinal samples and their related abdominal samples. The last group containing the variables *Moraxellaceae* (C), *Acinetobacter* (D), *Psychrobacter* (E), *Flavobacterium* (F) and *Chryseobacterium* (G) were revealed as the distinguishing features of the storage ice (Fig. 7). Several of the skin mucus and abdominal samples were also drawn towards these variables, but they were not as tightly associated with the variables as the ice samples. There was no association between the storage ice and the intestinal samples. The green digestive tract samples were clustered tightly as they were dominated by only two variables. The yellow ice samples were also grouped, albeit more dispersed as their position in the plot was influenced by several variables. On the other hand, the purple skin mucus samples and the red abdominal samples were more scattered along both PC1 and PC2.

Weighted (Fig. S3A) and unweighted (Fig. S3B) UniFrac beta diversity calculations were also made for comparison and were in agreement with the principal component analysis. They also showed that the abdominal samples from storage box no. 3 grouped together with the digestive tract samples at one end of the PC1 axis, explaining ∼72% of the variation in the samples (Fig. S3A). The other abdominal samples were more mingled together with the skin mucus samples further along the PC1 axis and the ice samples were situated furthest away from the digestive tract samples. A PERMANOVA test of the significance of sample groupings according to sample type using the weighted UniFrac distance matrix and based on 999 permutations proved significant (*F* = 13.82, *p* = 0.001).

### Gelatinase activity

**Figure 8:**
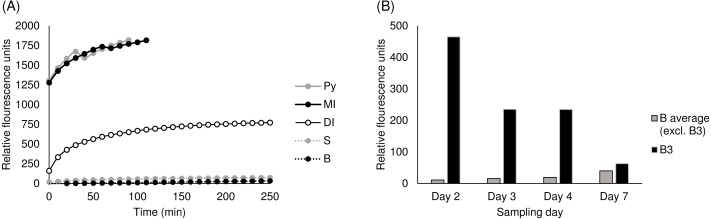
Gelatinase activity in (A) various sample types and in (B) abdominal samples. (A) Gelatinase activity in various representative samples from salmon taken on day 2. DI, distal intestine, MI, mid intestine, PY, pyloric caeca, Oe, Oesophagus, S, skin mucus, B, abdominal cavity. (B) Gelatinase activity in all samples taken from the abdominal cavity over a seven days period post slaughter. B3, abdominal cavity samples from storage box no. 3, B average, abdominal cavity samples from all storage boxes other than no. 3.

The initial screening comparing the various sample types showed that gelatinase activity (Fig. 8A) in the skin mucus and abdominal cavity was low and had a slow linear growth similar to the negative control and was thus consistent with the absence or near absence of enzyme activity.

On the other hand, samples from the pyloric caeca and mid intestine had a very high activity of gelatin degrading enzymes as the flourescence was high already after a few minutes of incubation. The distal intestine also showed gelatinase activity, although lower than the pyloric caeca and the mid intestine. The further analysis of all samples revealed that there was high enzymatic activity in the abdominal samples taken from the salmon in storage box no. 3. This was significantly different from the abdominal samples from the salmon in the other five storage boxes (Fig. 8B) (*Q* = 9.43, *p* = 0.001), where the measured gelatin degradation was consistent with the findings in the initial screening (Fig. 8A). The high enzyme activity in storage box no. 3 decreased over time and on day 7 was almost as low as in the other storage boxes. The skin mucus samples had very low gelatinase activity consistent with the initial screening.

## Discussion

The alpha diversity values for the digestive tract samples were consistent with previous studies on salmon intestinal microbiota (Gajardo et al., 2016; Dehler, Secombes & Martin, 2017). The high relative content of *Mycoplasmateaceae* detected in the samples from the digestive tract (Fig. 3B) is also consistent with other studies also based on salmon living in seawater (Llewellyn et al., 2016; Karlsen et al., 2017) although previous studies on the salmon intestinal microbiota have reported *Proteobacteria*, *Bacteriodetes*, *Firmicutes* and *Tenericutes* as the main phyla detected (Gajardo et al., 2016; Llewellyn et al., 2016; Dehler, Secombes & Martin, 2017). *Mycoplasmataceae* is a heterogeneous group of small bacteria lacking a cell wall and inhabiting a wide range of hosts as part of a parasitic lifestyle. Although *Mycoplasmataceae* includes many pathogens, they seem to be a commensal part of the intestinal microbiota of salmon (Llewellyn et al., 2016). The genus *Aliivibrio,* which was relatively abundant in the samples from the digestive tract, was also previously detected at relatively high levels in the digestive tract of sea-farmed salmon (Green, Smullen & Barnes, 2013; Karlsen et al., 2017). *Aliivibrio* consists of marine bacteria some of which are mutualistic, symbionts or pathogens in a range of marine animals including salmon (Beaz-Hidalgo et al., 2010). The similarity in bacterial community composition detected in this study is similar to that reported by some studies while others have found more diversification along the digestive tract (Gajardo et al., 2016; Egerton et al., 2018).

To our knowledge no previous studies have included samples from the abdominal cavity and direct comparison is therefore not possible. The clear contrast between the bacterial composition in abdominal fluids taken from salmon in storage box no. 3 and the other storage boxes indicated that the bacterial communities had different origins. The dominating *Mycoplasmataceae* likely originated from the digestive system as the relative content of *Mycoplasmatacea* in the abdominal samples from storage box no. 3 was no different than that of the digestive tract samples. The beta diversity analysis (Fig. 7) also indicated that *Mycoplasmataceae* and *Aliivibrio* originated from the digestive system and supported the suggestion of transfer of bacterial communities from the gastrointestinal tract to some of the abdominal samples, mainly those from storage box no. 3. The presence of *Enterobacteriaceae, Elizabethkingia,* and other marine and environmental bacteria, such as the genus *Janthinobacterium* and the family *Moraxellaceae*, in the abdominal samples from salmon in the other five storage boxes, indicated that these bacterial communities more likely originated from the seawater and the exterior surfaces of the salmon, or from the industrial water used in processing the salmon. *Photobacterium*, which was relatively abundant in some of these five storage boxes, consists mainly of marine bacteria that can inhabit both outer surfaces as well as the intestines of various marine fish species, including Atlantic salmon (Urbanczyk, Ast & Dunlap, 2011). The differences detected between sampling days in samples from the same storage box might be a reflection of shifts in the compositions of the bacterial communities, which is natural especially for perturbed environments (Gerber, 2014) such as the abdominal cavity of newly slaughtered salmon. The co-occurrence of *Enterobacteriaceae, Janthinobacterium,* and *Elizabethkingia* at their highest relative abundances on day 3 and 7 while all were detected at low relative abundances on day 2 and 4 might suggest a a mutualistic relationship between them and/or a common intolerance of certain other bacteria.

The microbial community in the skin mucus had a relatively even OTU abundance distribution (Fig. 5B) and relatively high Chao1 and observed OTU estimates. Other studies have reported a lower level of Chao1 estimates but higher phylogenetic diversity in skin microbiota of Atlantic salmon (Lokesh & Kiron, 2016). Analysis of skin microbiota in other fish species have shown lower levels of observed OTUs and lower or similar levels of phylogenetic diversity (Chiarello et al., 2015; Lowrey et al., 2015), although with fewer reads. However, the different sampling and storage conditions have to be taken into account when comparing the skin mucus microbiota detected in this study with that found in other studies. We have in this study not investigated to what degree the slaughtering and cleaning procedure has affected the bacterial composition detected, but others have also reported high diversity and even distribution of OTU abundances in skin mucus of various fish species (Chiarello et al., 2015; Lowrey et al., 2015). In general, the bacteria presently detected at high abundances did not correlate well with previous studies of the skin mucus of salmon (Lokesh & Kiron, 2016; Minniti et al., 2017), but this might also be explained by the different experimental setups and sampling conditions in the different studies, in particular by the industrial rinsing of the fish in our study and the potential mixture of bacterial communities originating from the intestines.

The bacterial community structure in the skin mucus from salmon in storage boxes no. 1, 2, and 3 had similar features, while the bacterial community in skin mucus from salmon in the other three boxes seemed to have other characteristics. This suggested an effect of the cleaning procedure. *Proteobacteria* has been reported as the overall dominant phylum present in the skin microbiota of salmon living in a marine environment (Minniti et al., 2017) and one possible explanation for the lower content of *Proteobacteria* in the skin mucus of salmon in storage boxes no. 1, 2, and 3 might be that more of the *Proteobacteria* has been washed away or further diluted with presumably dead bacteria by the additional industrial fresh water rinsing. In addition, the relative content of *Mycoplasmataceae* varied a great deal between the samples and ranged from less than 0.01% to 76.29%. *Mycoplasmataceae* has not previously been mentioned as a normal part of the skin mucus microbiota (Chiarello et al., 2015; Lowrey et al., 2015). It is possible that the *Mycoplasmataceae* detected in the skin mucus in this study might again be due to transfer from the intestines during slaughter. Therefore, in contrast to the abdominal fluids, the extra rinsing seemed to reduce the potential presence of intestinal fluids on the skin mucus. In addition, the predominantly marine bacterial genus *Photobacterium,* containing several species associated with fish, was also detected at higher abundances in the skin mucus of salmon not rinsed an extra time. Most of the remaining relatively abundant bacteria found in the skin mucus, like *Janthinobacterium*, *Chryseobacterium* and *Elizabethkingia*, and *Moraxellaceae* are common in freshwater as well as in the marine environment. In addition, *Staphylococcus*, which on average represented 6.4% of the bacterial community detected in the skin mucus, is commonly detected on skin and mucous membranes of various organisms. Also *Enterobacteriaceae*, which represented on average 6.0% of the skin mucus microbiota, and the order *Lactobacillales* contain numerous species of bacteria found widespread in nature. The multivariate analysis (Fig. 7) indicated that *Elizabethkingia, Enterobacteraceae*, and *Janthinobacterium* mainly originated from the marine environment or salmon exterior as they were not associated with intestinal or storage ice samples but with some of the skin mucus and abdominal samples. The PC plot also indicated that the skin mucus and abdominal samples had varying degrees of correlations with a multitude of bacteria, including the ten variables shown as well as the other 355 bacterial groups used in the multivariate analysis.

Although the relative content of *Moraxellaceae* in the skin mucus of salmon from storage boxes no. 1, 2, and 3 and storage boxes no. 4, 5, and 6, was not statistically different (*F* = 3.84, *p* = 0.062), 40% of the skin mucus samples from storage boxes no. 4, 5, and 6 contained >5% of *Moraxellaceae* (5.3% –27.4%) while only 10% of the skin mucus samples from storage boxes no. 1, 2, and 3 contained >5% (6.2%). Therefore, the difference in *Moraxellaceae* content in the storage ice might be caused by the skin mucus bacterial composition. On the other hand, the relative content of the known psychrotrophic genus in *Moraxellaceae, Psychrobacter,* did not increase. The reason for this counter-intuitive pattern might be that *Psychrobacter* was detected at relatively low levels only in the skin mucus. Therefore, the source of these bacteria might have been the water and/or storage ice. Because the water and ice used in the slaughtering facility is UV-treated the bacteria were likely dead and therefore either remained the same or diminished in comparative abundance while other living bacteria were transferred to the ice and could grow in abundance. This would further suggest that the unidentified *Moraxellaceae* in the slush ice originated from elsewhere than the industrial water, and most likely from the skin mucus.

Because *Acinetobacter* was detected at relatively low levels in the skin mucus of all salmon, the reason for the increase in relative abundance in the ice of storage boxes no. 1, 2, and 3 only is uncertain, but might be because the increase of the unidentified *Moraxellaceae* in the ice in storage boxes no. 4, 5, and 6 either hampered or camouflaged any increase in *Acinetobacter.* Four other bacterial genera also detected at relatively high abundances, *Janthinobacterium*, *Micrococcaceae, Chryseobacterium*, and *Flavobacterium* (fam. *Flavobacteriaceae*), are widespread in nature and could originate from the freshwater used during processing or the storage ice as well as be transferred from the salmon skin mucus, where they also were detected. Overall, the bacterial composition in the storage ice changed more than the other sample types during the sampling period, and seemed partly influenced by the skin mucus microbiota of the salmon. The PC plot (Fig. 7) also suggested that *Moraxellaceae, Acinetobacter, Psychrobacter, Flavobacterium*, and *Chryseobacterium* either originated from the storage ice or were transferred from the exterior of the salmon. In addition, the high phylogenetic diversity values of the storage ice samples might be due to a mixture of microbiota originating from the fish and microbiota killed by UV treatment but still present in the industrial water that the storage ice was made from. *Mycoplasmataceae* was detected only at low levels in the storage ice at the first sampling day with a maximum of 3.9% and further decreased the following days, indicating that the transfer of intestinal material to the storage ice was minor or that their survival in the ice slush without the immediate contact with the fish was minimal. This was supported by the multivariate analysis which showed no correlation between *Mycoplasmataceae* and the storage ice samples.

The measurements of gelatinase activity clearly demonstrated that the digestive tract samples contained enzymes capable of degrading gelatin. The DQ gelatin can be degraded by several enzymes including gelatinases such as MMP 2 and MMP 9 (Gill & Parks, 2011), and therefore this was not a measurement of a specific enzyme but rather the collective gelatin degrading activity in the samples. The results indicated a source of gelatinase activity in the abdominal cavity of the salmon in storage box no. 3 not present in the other boxes. The previous finding that all the abdominal samples from storage box no. 3 also contained a bacterial community structure highly similar to those in the digestive tract samples suggests that the high gelatinase activity may be due to enzymes originating from the intestinal fluids. The bacterial family *Mycoplasmataceae,* which was the dominating OTU detected in both intestinal samples and abdominal samples from storage box no. 3, contains several gelatinase producing bacteria in the genus *Mycoplasma* (Czekalowski, Hall & Woolcock, 1973). Therefore, the high gelatinase activity detected might be due to bacterial activity. However, a few other abdominal samples also had high relative abundances of *Mycoplasmataceae* without showing high gelatinase activity. The absolute amount of bacteria present was not estimated in this study, but might of course be of importance in relation to the gelatinase activity measurements. Because *Mycoplasma* can grow in intestinal fluids and blood, the presence of these in the abdominal cavity post-slaughter might also be a contributing factor. In addition, blood and intestinal fluids can contain gelatinases and other MMPs produced by the salmon (Hauser-Davis, Lima & Campos, 2012; Eysturskarð et al., 2017). The decreasing activity in the abdominal samples from storage box no. 3 may be due to the gradual inactivation of enzymes introduced at slaughter from either blood, intestinal fluids, intestinal bacteria, or a mixture thereof. In contrast, the slow increase in gelatinolytic activity detected in the other five storage boxes could possibly suggest a growth of other bacteria capable of degrading gelatin. Other genera detected at low relative abundance in most abdominal samples, such as *Staphylococcus, Bacillus* (fam. *Bacillaceae*)*, Pseudomonas* (fam. Pseudomonadaceae)*,* and *Clostridium* (fam. Clostridiaceae) contain species with gelatinolytic capabilities (Whaley et al., 1982; Chakraborty, Mahapatra & Roy, 2011; Balan et al., 2012; Zhang et al., 2015; Abed et al., 2016).

## Conclusions

A correlation was detected between the bacterial community composition and the gelatinase activity in the abdominal cavity of the salmon during cold storage. The bacterial composition in the intestines was highly dominated by *Mycoplasmataceae* and to a lesser degree *Aliivibrio*. The same dominance of *Mycoplasmataceae* was detected in the abdominal samples from storage box no. 3, while the abdominal samples from the other five storage boxes had a significantly different and more diverse bacterial community structures. The multivariate analysis grouped the abdominal samples from storage box no. 3 together with the intestinal samples. In addition, the gelatinase activity in the abdominal samples from storage box no. 3 was significantly higher than in the abdominal samples from the other storage boxes. At the same time the gelatinase activity was highest in the intestinal samples. This indicated the presence of intestinal fluids and bacteria in the abdominal cavity of salmon in storage box no. 3 and a possibility of connective tissue degradation as a consequence. This knowledge provides the industry with an incentive to be meticulous with the cleaning procedure and potential methods to use in quality control thereof.

The gelatinase activity in the skin mucus was low throughout. The relative content of *Mycoplasmataceae* varied but was generally low in the skin mucus and storage ice samples. The microbiota in the skin mucus was highly diverse and contained a mixture of bacteria likely stemming from both the marine environment and the industrial water used in the slaughtering facility. The relative content of *Firmicutes* was significantly higher in the skin mucus samples from salmon rinsed an extra time while *Proteobacteria* was significantly lower in these samples. The microbial community in the storage ice had significantly higher phylogenetic diversity than the other sample types. Potentially, the storage ice samples might have contained various bacteria common in freshwater as well as bacteria originating from the skin mucus.

##  Supplemental Information

10.7717/peerj.7040/supp-1 Figure S1 Rarefaction curves Rarefaction curves for all samples of (A) Chao1, (B) Observed OTUs and (C) PD whole tree alpha diversity estimates.

10.7717/peerj.7040/supp-2 Figure S2 Group-wise rarefaction curves Mean rarefaction curves with standard deviation of (A) Chao1, (B) Observed OTUs, and (C) PD whole tree alpha diversity estimates for all sample types.

10.7717/peerj.7040/supp-3 Figure S3 PC plots of beta diversities PC plots of (A) weighted and (B) unweighted UniFrac calculations. Each coloured circle represents a sample. Red: abdominal samples, purple: skin samples, yellow: ice samples, green/blue/orange: digestive tract samples. The grouped ice samples and digestive tract samples are encircled in both plots. The encircled red abdominal samples are those from storage box no. 3.

10.7717/peerj.7040/supp-4 Table S1 OTU table

10.7717/peerj.7040/supp-5 Table S2 Gelatinase activity measurements

## Additional Information and Declarations

### DNA Deposition

The following information was supplied regarding the deposition of DNA sequences: The sequence data supporting the conclusions of this article is available in the NCBI Sequence Read Archive database, study accession no.: SRP149649.

### Data Availability

The following information was supplied regarding data availability: A complete OTU table is available in Table S1. The gelatin degrading activity measurements are available in Table S2.
