# Supplementary figures and images for: Composition and dynamics of the bacterial communities present in the post-slaughter environment of farmed Atlantic salmon (Salmo salar L.) and correlations to gelatin degrading activity
Source: PeerJ. 2019 Jun 4;7:e7040. doi: 10.7717/peerj.7040 (PMC6555393; doi:10.7717/peerj.7040)

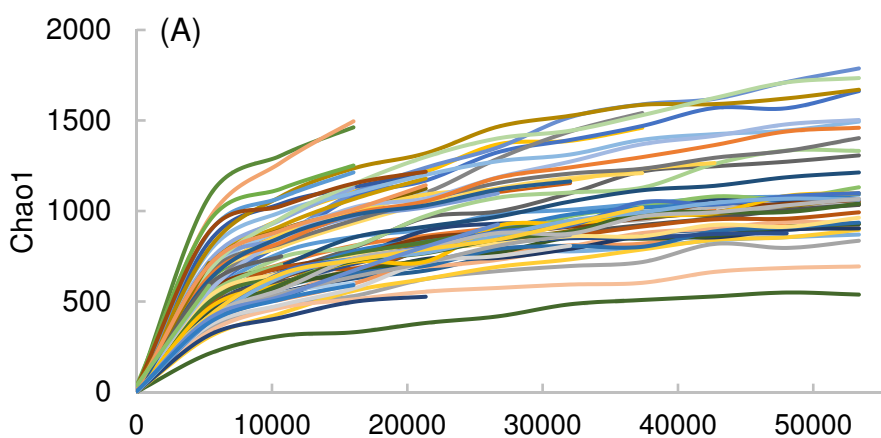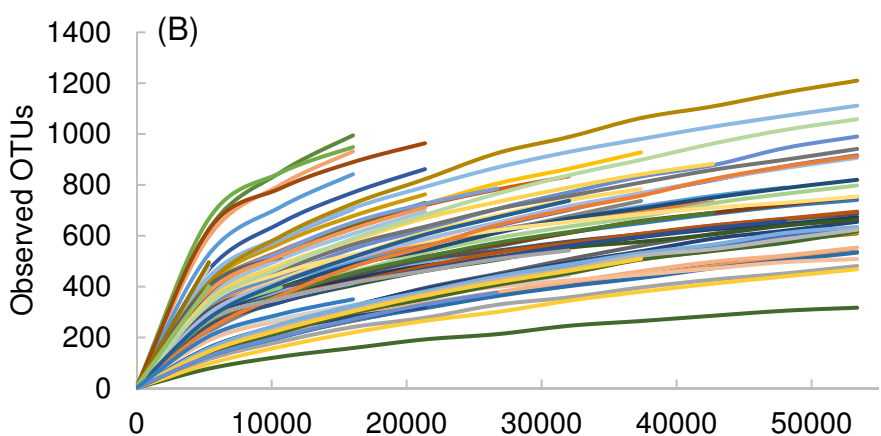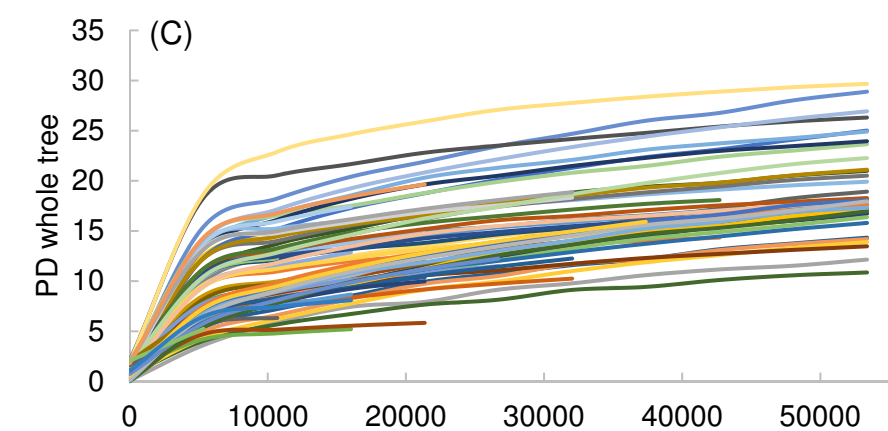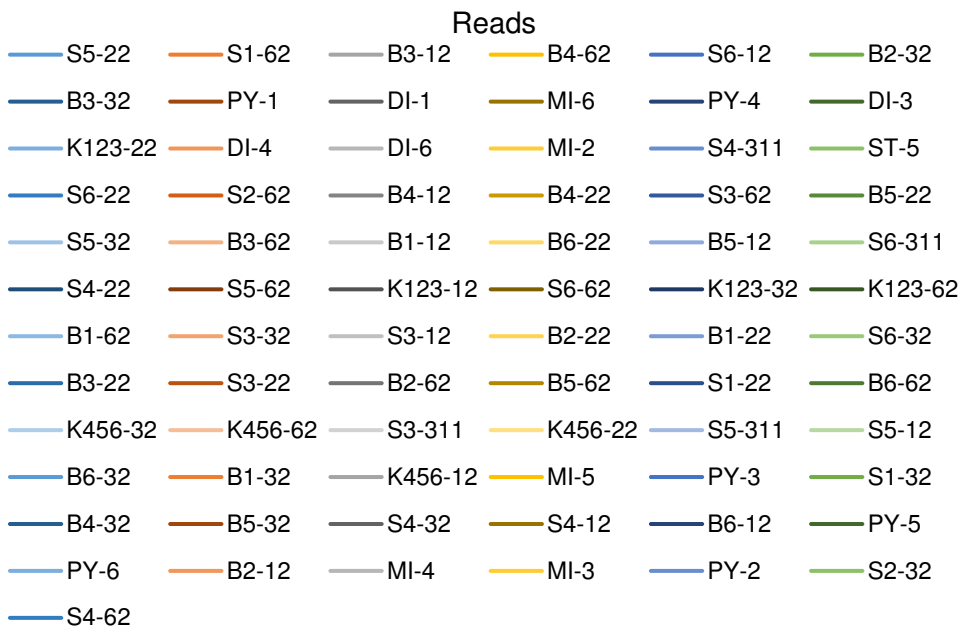

Supplement: Figure S1 — Rarefaction curves for all samples of (A) Chao1, (B) Observed OTUs and (C) PD whole tree alpha diversity estimates. [file peerj-07-7040-s001.pdf]

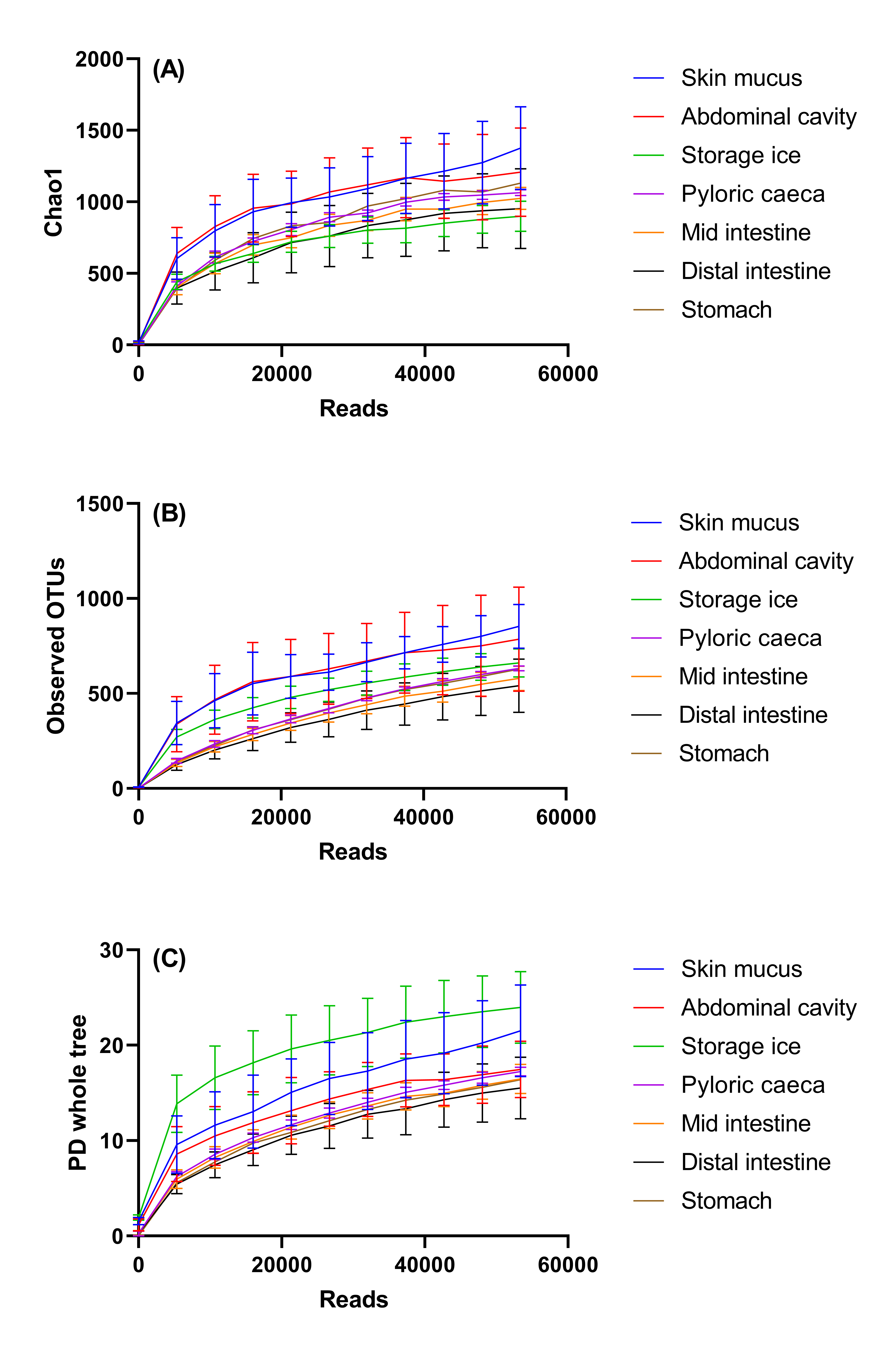

Supplement: Figure S2 — Mean rarefaction curves with standard deviation of (A) Chao1, (B) Observed OTUs, and (C) PD whole tree alpha diversity estimates for all sample types. [file peerj-07-7040-s002.png]

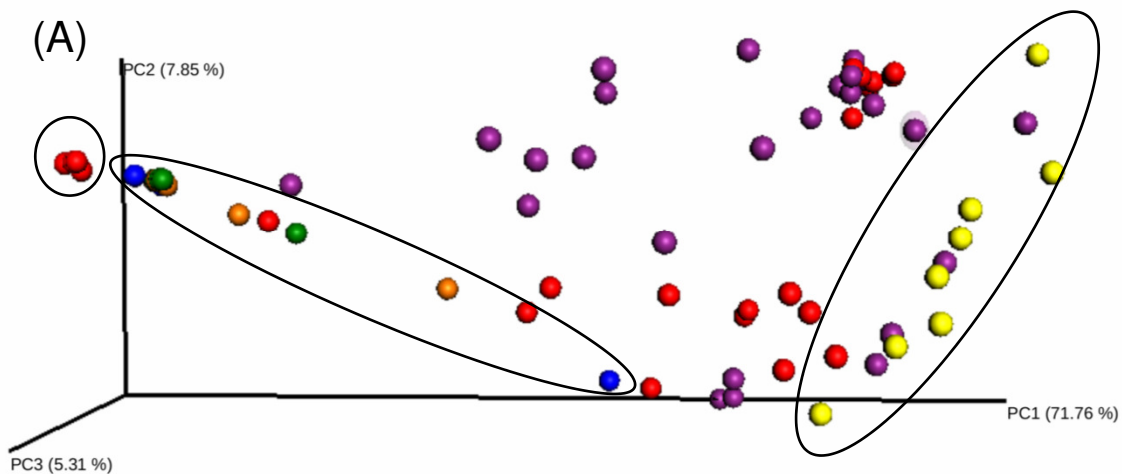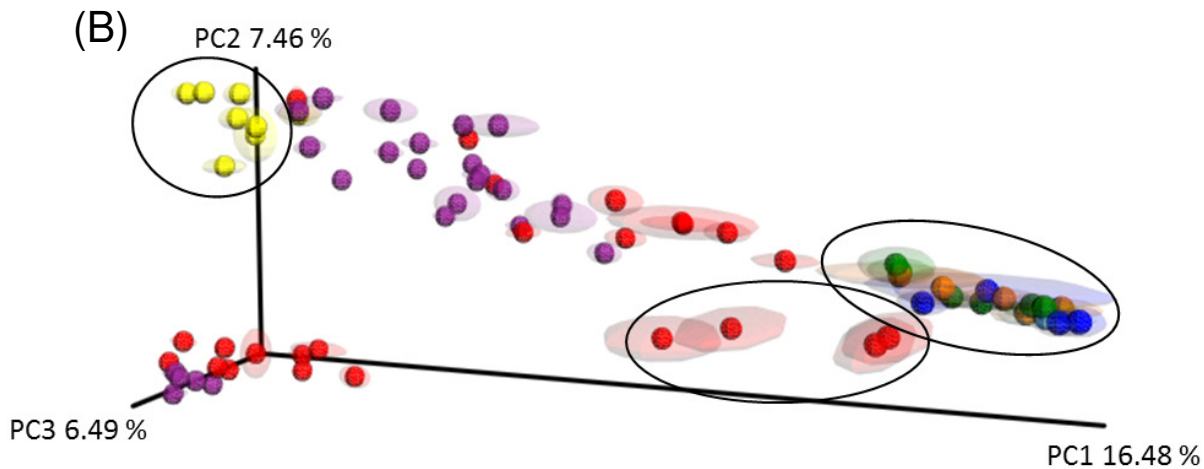

Supplement: Figure S3 — PC plots of (A) weighted and (B) unweighted UniFrac calculations. Each coloured circle represents a sample. Red: abdominal samples, purple: skin samples, yellow: ice samples, green/blue/orange: digestive tract samples. The grouped ice samples and digestive tract samples are encircled in both plots. The encircled red abdominal samples are those from storage box no. 3. [file peerj-07-7040-s003.pdf]
